# Clinical Implementation of a Fully Digital Workflow for the Fabrication of a Maxillary Complete Denture: A Case Report

**DOI:** 10.3390/dj13110524

**Published:** 2025-11-10

**Authors:** Carlos Roberto Luna-Domínguez, Ana Cecilia Luna-Vega, Marco Felipe Salas-Orozco, Rogelio Oliver-Parra, Carlos Alberto Luna-Lara, Jorge Humberto Luna-Domínguez

**Affiliations:** 1 Faculty of Dentistry, Autonomous University of Tamaulipas, Av. Universidad Esq. con Blvd, Adolfo Lopez Mateos S/N, Tampico 89337, Tamaulipas, Mexico; cldominguez@uat.edu.mx (C.R.L.-D.); roliver@docentes.uat.edu.mx (R.O.-P.); cluna@docentes.uat.edu.mx (C.A.L.-L.); 2 Clinical Research Laboratory, Faculty of Stomatology, Autonomous University of San Luis Potosi, San Luis Potosi 78290, San Luis Potosi, Mexico; marcos-salas@hotmail.com

**Keywords:** complete denture, digital workflow, CAD/CAM, maxillary edentulism, 3D printing, intraoral scanning, prosthodontics, PMMA

## Abstract

**Background/Objectives:** Edentulism is a prevalent chronic condition among older adults, and conventional complete dentures remain the standard of care. However, their fabrication often involves multiple clinical sessions and operator-dependent steps that may compromise fit and comfort. Digital workflows using CAD/CAM technologies have emerged as viable alternatives, offering improved efficiency, precision, and patient-centered outcomes. This case report aims to present a fully digital workflow for maxillary complete dentures and describe clinical efficiency and patient-reported outcomes. **Case Presentation:** A 73-year-old edentulous male patient underwent maxillary rehabilitation using a fully digital workflow. The protocol included intraoral scanning; the design and 3D printing of a custom tray with occlusal rims; border-molded functional impressions; virtual articulation; and CAD/CAM fabrication. A digitally designed Try-In denture was 3D printed for clinical evaluation, followed by adjustments. The definitive prosthesis was milled from high-performance PMMA discs using a five-axis milling machine. The workflow reduced the number of appointments and laboratory steps. At six-month follow-up, the patient reported high satisfaction with esthetics, retention, phonetics, and masticatory performance. No significant post-delivery adjustments were required. **Conclusions:** This case demonstrates that fully digital workflows for maxillary complete dentures are clinically viable, providing excellent precision, patient satisfaction, and time efficiency compared to conventional methods. The reproducible protocol described may support the broader integration of CAD/CAM technologies in edentulous rehabilitation.

## 1. Introduction

Edentulism remains a common chronic condition, affecting an estimated 4% of the world’s adult population and more than one-third of individuals ≥75 years of age [1]. Conventional complete dentures (CDs) have long served as the primary treatment; however, their fabrication involves multiple appointments, operator-dependent laboratory steps, and polymerization shrinkage that can compromise fit, retention, and long-term comfort [2]. In the past decade, computer-aided design and computer-aided manufacturing (CAD/CAM) technologies have emerged as an alternative workflow for complete denture fabrication. Milled or printed dentures offer several theoretical advantages: fewer clinical visits, shorter chair time, stable pre-polymerized polymethyl methacrylate (PMMA) with superior mechanical properties, and the ability to archive digital design files for rapid remakes [3].

Current evidence supports the clinical viability of digitally fabricated CDs. A 2024 meta-analysis by Avelino et al. compared 346 patients across 16 trials and found that CAD/CAM dentures demonstrated denture base adaptation, retention, and clinician-rated quality scores comparable with or superior to conventional dentures. Patient-reported outcome measures (PROMs), including satisfaction and oral health-related quality of life, were statistically equivalent [4]. However, the review also highlighted substantial heterogeneity across studies, particularly in relation to impression techniques, jaw relation registration, and manufacturing methods, and emphasized the lack of standardized clinical and laboratory protocols.

Thu et al. corroborated this observation in a recent systematic review that mapped 21 workflow variations in digital denture fabrication. The authors concluded that “the high heterogeneity in digital complete denture workflows is attributable to the absence of established protocols for each procedural step” [5]. Although many clinical trials employ direct intraoral scans, current evidence shows that border-molded functional impressions provide significantly better retention (~14–16 N vs. ~6 N for intraoral scans alone). Moreover, few reports integrate functional impressions and virtual articulation within a single, end-to-end workflow [5]. Additionally, most published studies report outcomes over short-term follow-up periods (≤3 months), leaving a critical gap in medium-term clinical validation.

In this context, the present case report introduces a fully integrated digital protocol that merges the precision of CAD/CAM technology with the reliability of conventional impression techniques. The proposed approach is characterized by the following:

1. The use of a 3D-printed custom tray for capturing a border-molded functional impression, ensuring optimal mucosal extension and support.
2. Virtual articulation based on a rescanned tray–rim assembly, eliminating the need for facebow transfer while preserving a verified vertical dimension of occlusion.
3. Tooth arrangement performed in Exocad, guided by midline, canine, and smile references, followed by a 3D-printed Try-In for clinical evaluation.
4. Subtractive CAM fabrication using separate high-performance PMMA discs for the denture base and multilayer PMMA teeth, bonded with a dual-cure adhesive interface that meets ISO 22112:2017 standards for tooth retention [6].
5. A six-month follow-up assessing prosthesis adaptation, retention, and patient satisfaction without the need for unscheduled adjustment visits.

This clinical case report is, to the best of our knowledge, the first to document a fully digital workflow for maxillary complete dentures that integrates a border-molded functional impression, virtual articulation, and dual-disc milling of the base and teeth. The step-by-step methodology described here, combined with a six-month clinical follow-up, provides a clear and reproducible example of how digital and conventional techniques can be successfully merged. By detailing each phase of the protocol, from data acquisition to final delivery, this report helps address the current lack of standardization in CAD/CAM denture fabrication methods and supports ongoing efforts to establish clinically validated digital workflows. All procedures were conducted in accordance with the Declaration of Helsinki and received approval from the Institutional Review Board of the Faculty of Dentistry, Autonomous University of Tamaulipas (IRB: CEI-FO-UAT-014/2024 (093D 2024 03); approved on 21 March 2024).

## 2. Clinical Case

### 2.1. Patient Selection

**Figure 1:**
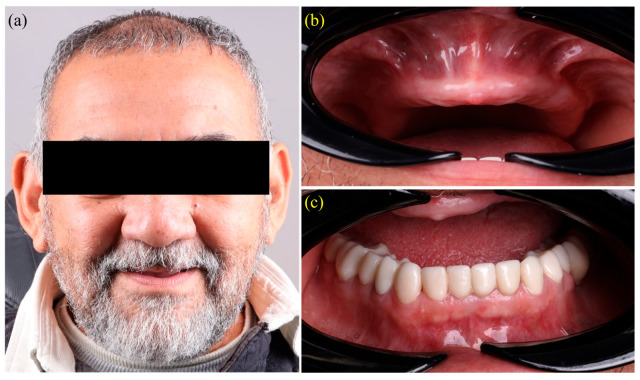
Baseline records: (**a**) frontal extra-oral view with ocular region masked; (**b**) completely edentulous maxillary ridge; (**c**) mandibular arch restored with a monolithic zirconia fixed dental prosthesis, providing the opposing occlusion for the planned fully digital maxillary complete denture.

A 73-year-old male with no relevant systemic conditions presented to the postgraduate prosthodontics clinic at the Universidad Autónoma de Tamaulipas for rehabilitation of a completely edentulous maxilla. The mandibular arch had previously been restored with a monolithic, metal-free zirconia fixed prosthesis (Figure 1a–c). Although a conventional complete denture could have fulfilled the patient’s functional needs, a fully digital workflow was selected instead. This approach was chosen to take advantage of the benefits offered by CAD/CAM technology, including improved fit accuracy, reduced chairside time, and patient-reported outcomes that are comparable or superior to those achieved with traditional methods [1,2]. The patient provided written informed consent for all clinical procedures, photography, and case publication. The protocol was approved by the Institutional Review Board of the Faculty of Dentistry, Autonomous University of Tamaulipas (IRB: CEI-FO-UAT-014/2024 (093D 2024 03); approved on 21 March 2024) and conducted in accordance with the Declaration of Helsinki.

### 2.2. Digital Maxillary Impression and 3D-Printed Custom Tray Fabrication

**Figure 2:**
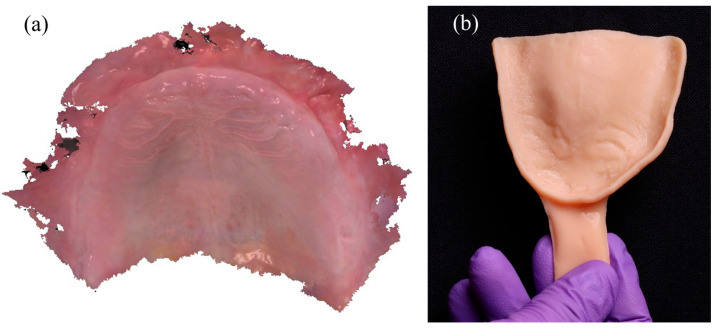
Digital maxillary scan and 3D-printed custom tray: (**a**) color PLY mesh of the edentulous maxilla acquired with a TRIOS 4 confocal intra-oral scanner; (**b**) patient-specific maxillary impression tray additively manufactured in Class IIa denture-base resin at a 45° build orientation for optimal dimensional accuracy.

Digital impressions of the edentulous maxilla were acquired using a confocal intraoral scanner (TRIOS 4, 3Shape A/S, Copenhagen, Denmark). This system combines confocal microscopy with ultrafast optical sectioning to accurately capture non-reflective, mobile mucosal tissues while minimizing distortion. This capability is particularly advantageous in fully edentulous arches, where tissue resilience and moisture can compromise scan accuracy [3,4]. The resulting color PLY mesh (Figure 2a) was exported and imported into DentalCAD 3.0 Galway (exocad GmbH, Darmstadt, Germany), where a custom impression tray was digitally designed. The tray was fabricated using a digital light processing (DLP) 3D printer (NextDent 5100, 3D Systems Inc., Soesterberg, The Netherlands) with a Class IIa biocompatible denture base resin and subsequently post-cured according to manufacturer recommendations (Figure 2b). A 45-degree build angle was selected to enhance dimensional accuracy and reduce staircase artifacts, especially in curved anatomical regions [7,8]. The tray was then disinfected and prepared for the border-molded functional impression.

### 2.3. Impression Recording and Maxillomandibular Registration

**Figure 3:**
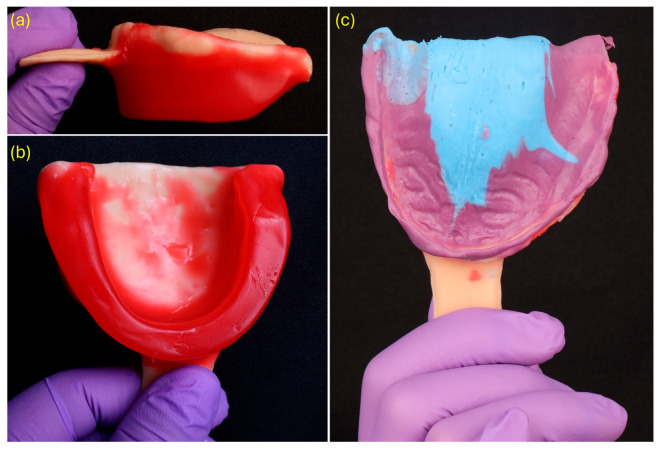
Wax rim fabrication and functional border molding: (**a**) lateral view of wax rim on printed tray (22 mm anterior/18 mm posterior); (**b**) occlusal aspect of the rim; (**c**) completed border molding using dual-viscosity PVS, with heavy-body material (purple) for the periphery and medium-body material (blue) for the intaglio surface.

A border-molded functional impression was obtained using the patient-specific tray previously fabricated. To establish the preliminary vertical dimension of occlusion (VDO) and ensure adequate perioral support, a wax occlusion rim was first adapted to the tray, measuring 22 mm in the anterior region and 18 mm in the posterior region (Figure 3a,b). The rim was then adjusted intraorally based on facial landmarks and lip dynamics prior to initiating the impression phase.

Heavy-body polyvinyl siloxane (PVS) (Aquasil Ultra+ Putty; Dentsply Sirona, Charlotte, NC, USA) was applied along the entire periphery of the custom tray to record the functional depth of the vestibular sulci. Subsequently, medium-body PVS (Aquasil Ultra+ Medium) was injected over the intaglio surface to capture the primary support areas under static pressure (Figure 3c). This dual-consistency technique allowed for a functional impression that accurately reproduced both compressible and non-compressible tissues [9,10]. Border-molded impressions have consistently shown superior retention in edentulous arches, achieving dislodgement forces of approximately 14–16 N compared to ~6 N obtained through intraoral scanning alone. For this reason, they continue to represent the reference standard in digital complete denture workflows [11].

**Figure 4:**
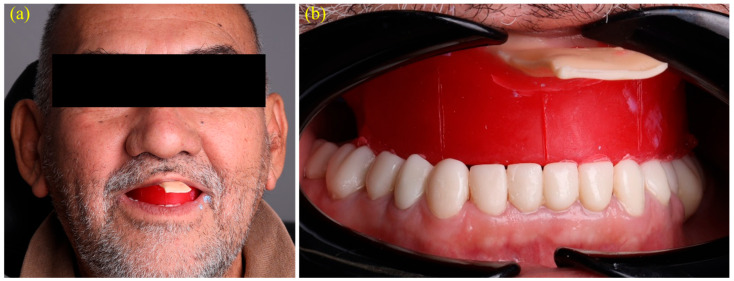
Transfer of anatomic reference lines for virtual tooth setup: (**a**) extra-oral verification of vertical dimension and facial midline with rim in situ; (**b**) intra-oral frontal view showing marked midline, canine guides, and smile line that will dictate the subsequent digital tooth arrangement.

**Figure 5:**
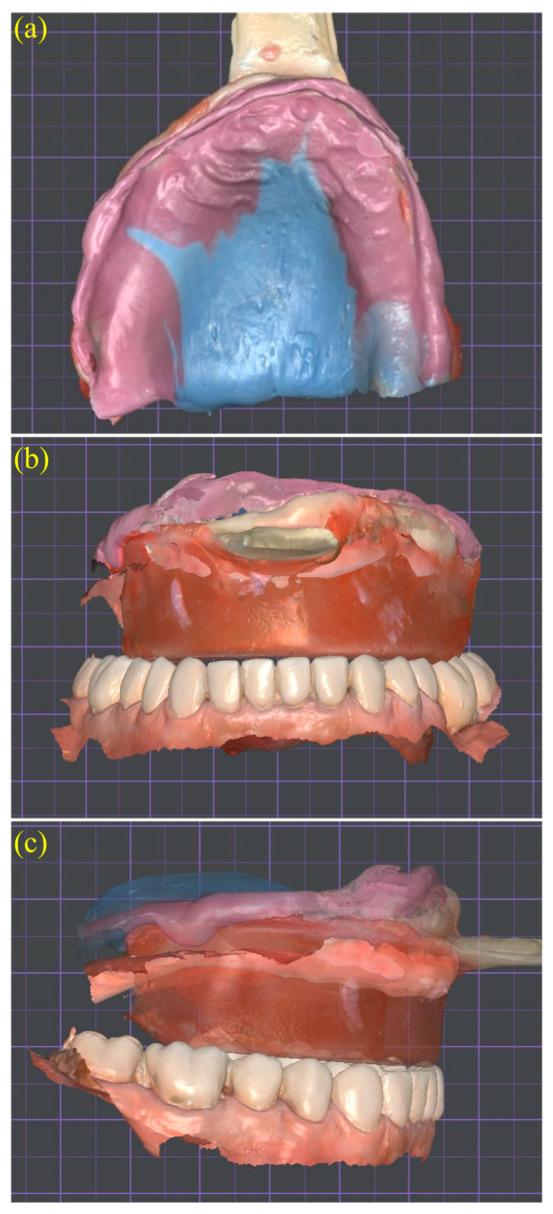
Digital articulation of maxillo-mandibular scans using the tray–rim reference: (**a**) maxillary color mesh showing functional impression material on the intaglio; (**b**) frontal and (**c**) right lateral views of superimposed maxillary and mandibular scans mounted in centric relation, preserving the verified vertical dimension and anteroposterior tooth position.

After the border-molding material had set, reference lines including the midline, canine guides, and smile line were drawn directly onto the wax rim to guide the subsequent digital tooth arrangement (Figure 4a,b). Once the vertical dimension of occlusion (VDO) and these anatomical landmarks were clinically verified, the entire tray and rim assembly was rescanned using a confocal intraoral scanner (TRIOS 4; 3Shape A/S, Copenhagen, Denmark). This scan captured both arches and the interocclusal record, generating color PLY meshes suitable for digital articulation (Figure 5a–c). Confocal optical sectioning technology minimizes tissue distortion during scanning and has been shown to produce trueness deviations below 0.2 mm when compared with conventional impression methods [10].

The merged scan served as a digital reference for articulating the maxillary and mandibular datasets within DentalCAD 3.0 Galway (exocad GmbH, Darmstadt, Germany), ensuring preservation of the verified vertical dimension of occlusion (VDO) and anteroposterior tooth position. This tray–rim-based articulation technique has demonstrated centric relation accuracy comparable to conventional facebow mounting in complete denture CAD/CAM protocols [12,13]. Because the scan captured both arches in occlusion with the functional impression in place, neither facebow transfer nor gothic arch tracing was necessary. This simplified approach aligns with current systematic review recommendations, which emphasize the importance of combining functional impressions with verified jaw relations to reduce the risk of occlusal discrepancies and unplanned post-insertion adjustments [11].

### 2.4. Virtual Tooth Arrangement and Try-In Design

The tray–rim assembly functioned as a digital reference for aligning the maxillary and mandibular scans in centric relation, allowing preservation of the clinically verified vertical dimension and anteroposterior tooth position. This approach eliminates the need for conventional transfer devices by using a scanned functional impression to define the occlusal relationship directly within the CAD environment. Recent studies have demonstrated that virtual articulation based on modified trays or dedicated intraoral devices can achieve a level of accuracy comparable to traditional mechanical methods in complete denture workflows [12,14].

**Figure 6:**
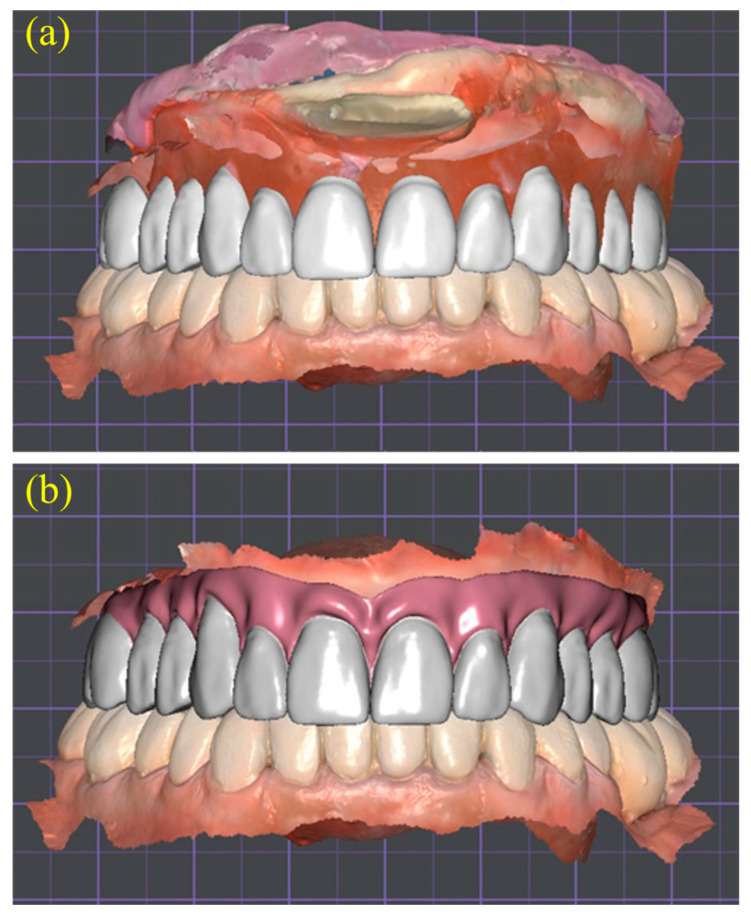
Virtual tooth arrangement generated using CAD software (DentalCAD 3.0 Galway, exocad GmbH, Darmstadt, Germany). (**a**) Preliminary placement of artificial teeth on the articulated arches; (**b**) finalized maxillary set-up with digitally sculpted gingival contours, providing three-dimensional assessment of esthetics, phonetics, and balanced occlusion prior to Try-In fabrication.

Using the articulated datasets, a virtual tooth arrangement was performed within the DentalCAD environment to generate a Try-In prototype. The artificial teeth were positioned according to the anatomic reference lines previously transferred to the occlusion rim, including the midline, canine guides, and smile line. This allowed for a three-dimensional evaluation of esthetics, phonetics, and occlusal balance before definitive fabrication (Figure 6a,b). Incorporating this digital Try-In step minimized the need for multiple conventional wax Try-Ins and facilitated more efficient communication between the clinical and laboratory teams [10,14].

### 2.5. Printed Try-In Evaluation and Functional Bite Registration

**Figure 7:**
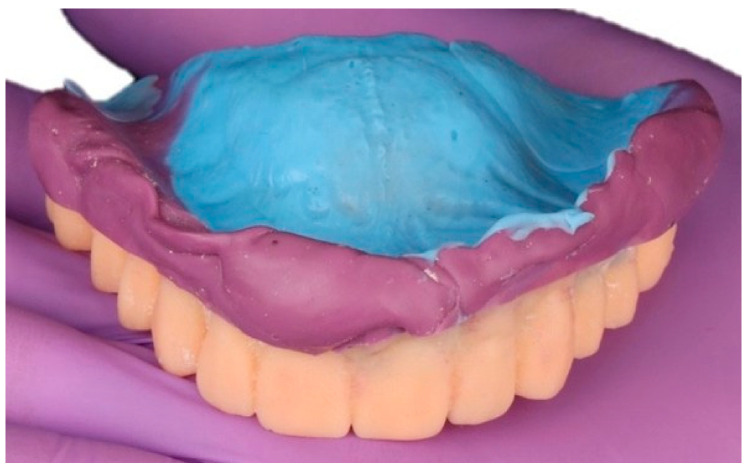
Try-In stabilized for definitive bite registration. Dual-viscosity PVS applied to 3-D printed Try-In: heavy-body material (purple) on borders, regular-body material (blue) on intaglio, enabling simultaneous capture of functional extensions and basal-seat detail while maintaining vertical dimension.

The provisional denture base and teeth were additively manufactured in a Class IIa biocompatible photopolymer and inserted intraorally for clinical evaluation. During the Try-In appointment, minor occlusal and esthetic adjustments were made to optimize phonetics, lip support, and the maxillomandibular relationship. To accurately capture the definitive interarch record, the adjusted Try-In was stabilized using a dual-viscosity polyvinyl siloxane (PVS) technique. Heavy-body material was applied to the periphery to record the functional extensions, while regular-body material was injected onto the intaglio surface to register the basal seat (Figure 7). This approach allowed for simultaneous registration of soft tissue detail and occlusal position, while maintaining the verified vertical dimension of occlusion [12]. The corrected Try-In thus served both as a diagnostic tool for intraoral validation and as a transfer device for precise virtual articulation in the subsequent digital workflow.

### 2.6. High-Resolution Rescanning and Digital Articulation

**Figure 8:**
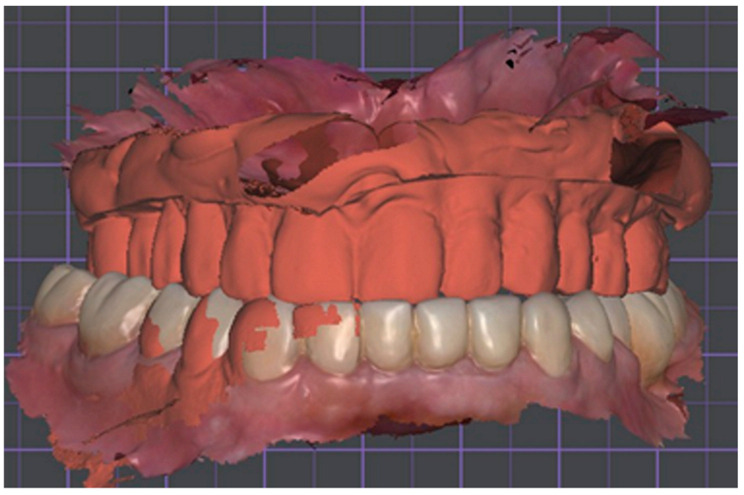
Digital superimposition of validated Try-In. Rescanned Try-In (opaque orange) overlaid on initial color meshes, confirming centric relation and vertical dimension prior to definitive CAD design.

**Figure 9:**
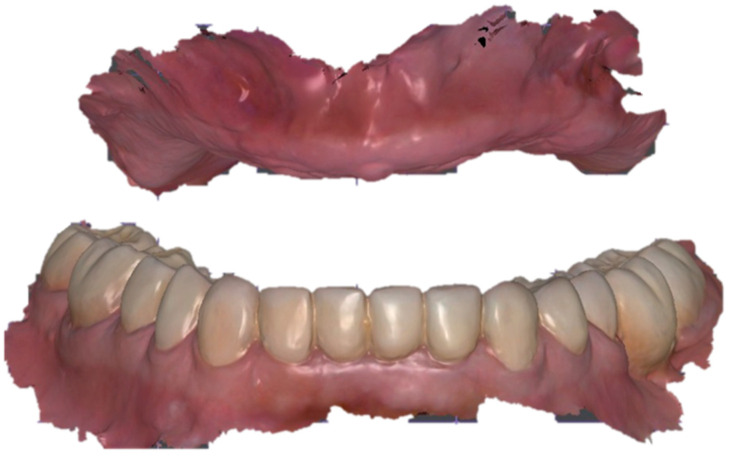
Extended soft-tissue capture after rescanning. High-resolution color meshes of maxilla (upper) and mandible (lower) displaying vestibular sulci and post-palatal seal, providing enhanced border data for the final denture base.

Following clinical approval of the Try-In, both arches were rescanned using a confocal intraoral scanner (TRIOS 4, 3Shape A/S, Copenhagen, Denmark) to capture the adjusted occlusal relationship. The resulting color PLY files were imported into DentalCAD 3.0 Galway (exocad GmbH, Darmstadt, Germany). Superimposing the updated Try-In scan onto the original datasets allowed preservation of the verified vertical dimension, occlusal plane, and esthetic parameters (Figure 8). The high-resolution scan also recorded extended soft-tissue landmarks, including the vestibular sulci, posterior palatal seal, and mobile mucosa, providing enhanced anatomical detail for the denture borders and reducing the likelihood of post-delivery adjustments [15] (Figure 9).

### 2.7. Definitive Virtual Denture Design

**Figure 10:**
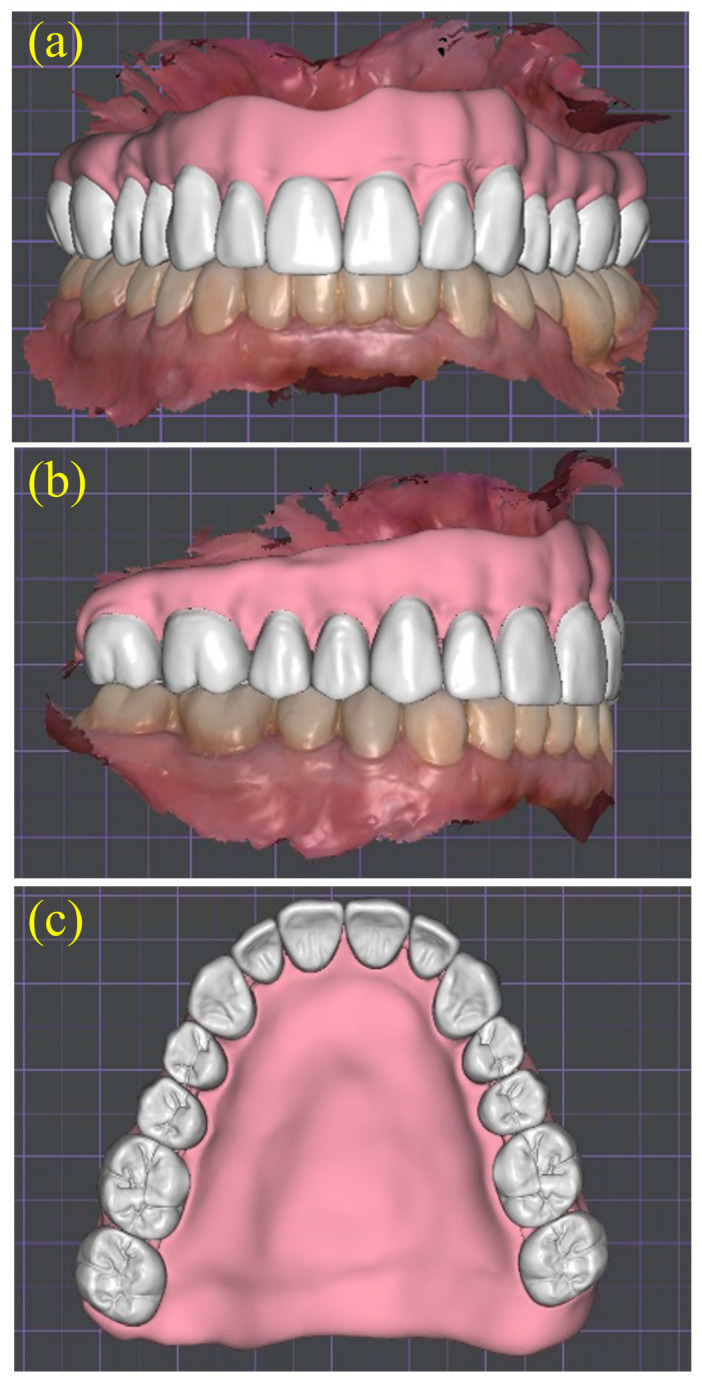
Definitive virtual denture design: (**a**) frontal, (**b**) right lateral, and (**c**) occlusal views of the finalized maxillary denture showing corrected tooth positions and digitally sculpted gingiva before CAM milling.

All clinical refinements identified during the Try-In phase, including corrections to the posterior crossbite and anterior edge-to-edge occlusal contacts, were incorporated into the final digital setup to optimize interarch balance and functional symmetry. Using the validated Try-In as a reference, the definitive virtual tooth arrangement was finalized in DentalCAD 3.0, ensuring appropriate incisal display, gingival contour, and bilateral occlusion (Figure 10a–c). The completed CAD design was then exported in STL format for subtractive manufacturing using a CAM protocol.

### 2.8. CAM Milling, Bonding, and Finishing

The definitive maxillary complete denture was fabricated using a subtractive computer-aided manufacturing (CAM) protocol. The final STL file was exported and transferred to a five-axis milling machine (DWX-52Di, Roland DGA Corp., Irvine, CA, USA). Two separate pre-polymerized PMMA discs were selected: one high-impact disc for the denture base and a multilayer chromatic disc for the denture teeth. This dual-disc approach enabled the use of highly cross-linked PMMA in the base to enhance fracture resistance and biocompatibility, while the stratified PMMA tooth disc provided improved color gradation, wear resistance, and esthetic integration [16,17].

**Figure 11:**
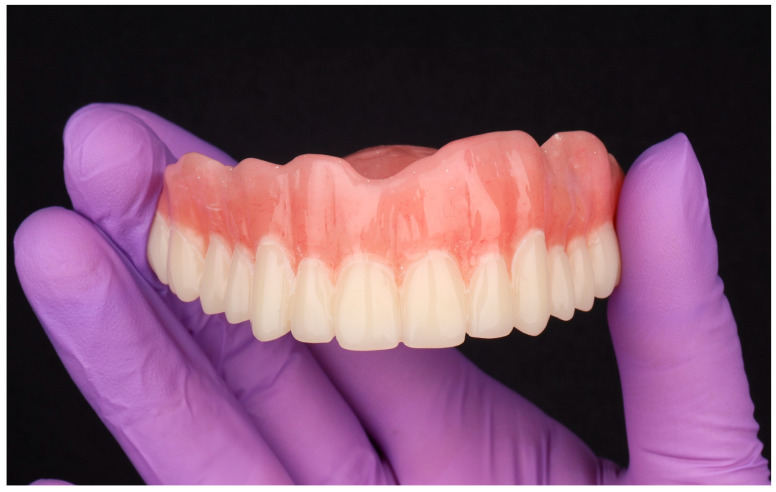
Definitive maxillary complete denture after subtractive CAD/CAM fabrication. The high-impact PMMA base and multilayer chromatic PMMA teeth were milled separately on a five-axis unit (DWX-52Di, Roland DGA Corp., Irvine, CA, USA), adhesively bonded with a dual-polymerizing MMA-based resin, and sequentially finished and polished to a high-luster surface (Ra < 0.2 µm).

Following the milling process, the denture base and teeth were joined using a dual-polymerizing methyl methacrylate (MMA)-based adhesive, creating a strong chemical–mechanical bond. This bonding protocol has demonstrated bond strengths exceeding 20 MPa, surpassing the minimum retention threshold established by ISO 22112 for denture tooth adherence [17]. After assembly, the prosthesis underwent surface characterization and sequential polishing using pumice and high-luster acrylic compounds, applied with rotary goat-hair and muslin wheels. This finishing protocol achieved a surface roughness below 0.2 µm, a value associated with reduced biofilm accumulation and improved long-term hygiene maintenance [18,19] (Figure 11).

### 2.9. Clinical Delivery and Six-Month Evaluation

**Figure 12:**
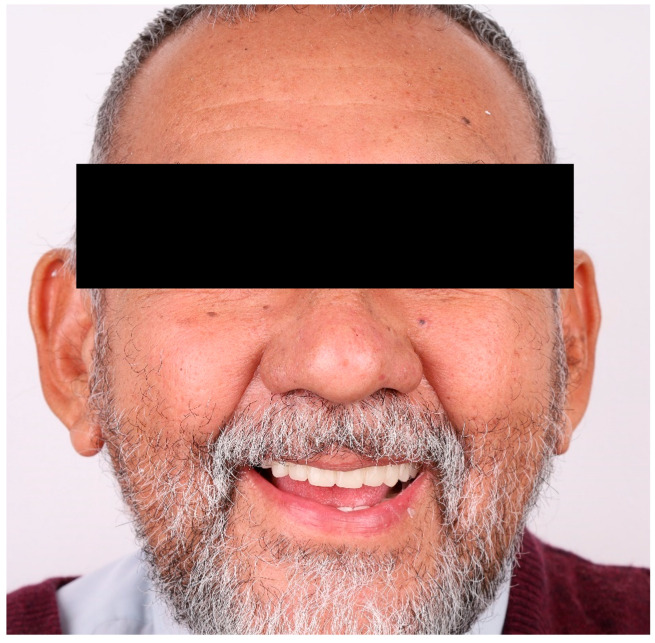
Six-month follow-up: frontal smile showing the maxillary complete denture fabricated using a fully digital subtractive CAD/CAM workflow. Clinical evaluation confirmed stable retention and healthy mucosa; no post-insertion adjustments were required.

The prosthesis was delivered without complications, and no occlusal or peripheral adjustments were necessary at the time of insertion. At the six-month follow-up appointment (Figure 12), the patient reported high levels of satisfaction with the denture’s comfort, esthetics, phonetics, and masticatory function. Clinical examination confirmed optimal adaptation, stable retention, and the absence of pressure spots or mucosal irritation. These findings are consistent with previous reports indicating that fully digital, subtractively milled complete dentures provide a predictable fit and reduce the need for post-insertion maintenance when compared to conventional fabrication methods [1].

## 3. Results

The definitive prosthesis was delivered without complications. No occlusal adjustments or peripheral reliefs were required at insertion. At the six-month follow-up (Figure 12), clinical evaluation confirmed excellent adaptation, stable retention, and the absence of mucosal irritation or pressure spots.

From the patient’s perspective, functional performance was rated consistently high. The ability to chew and swallow was described as effortless, and no discomfort was reported during speech or mastication. The patient expressed full satisfaction with the esthetic appearance of the teeth and gingival architecture, stating that the prosthesis felt natural and comfortable during social interactions. There were no dietary restrictions reported, and the patient did not experience any psychological discomfort or social avoidance due to the denture. Overall, patient-reported outcomes confirmed optimal comfort, function, and esthetic satisfaction.

These results support the effectiveness of the digitally guided workflow in delivering a clinically stable and patient-accepted complete denture with minimal post-insertion needs. The combination of border-molded impression accuracy, virtual articulation, and dual-disc subtractive manufacturing contributed to a predictable result with excellent clinical and emotional integration.

## 4. Discussion

This clinical case demonstrates the practical application of a fully digital workflow for the fabrication of a maxillary complete denture, emphasizing the integration of functional impressions, virtual articulation, and subtractive CAM technology. Rather than replacing conventional principles, the proposed protocol integrates their proven clinical value, such as border-molded impressions and occlusal validation, with the accuracy and efficiency offered by CAD/CAM systems. This combined approach provided full control over prosthetic parameters, reduced the number of clinical sessions, and led to a prosthesis that required no post-insertion adjustments. The result underscores how digital dentistry can successfully incorporate foundational prosthodontic concepts to enhance outcomes in edentulous rehabilitation.

A key differentiator in this case was the use of a 3D-printed custom tray specifically designed to accommodate border molding and vertical dimension registration. This step ensured accurate capture of soft tissue dynamics and anatomic landmarks, particularly in the posterior and vestibular regions. While many digital workflows rely exclusively on intraoral scans, recent evidence confirms that functional impressions continue to yield superior retention outcomes, especially when recording vestibular extensions and mucosal compressibility in fully edentulous arches [5]. By combining conventional impression protocols with digital planning, the clinician ensured optimal peripheral seal, which translated into a well-adapted prosthesis requiring no clinical adjustments at delivery.

The digital Try-In played a pivotal role in the workflow, enabling real-time intraoral validation of tooth position, esthetics, and phonetics. The patient was actively involved in approving the arrangement, and necessary occlusal refinements were incorporated before final milling. As supported by Avelino et al. [4], the inclusion of a digital Try-In phase contributes to improved patient-reported outcomes and reduces the incidence of remakes and functional errors.

For the definitive prosthesis, the use of a dual-disc subtractive manufacturing approach was both biologically and mechanically advantageous. The base was milled from a high-impact pre-polymerized PMMA blank, while the denture teeth were fabricated from a multilayer chromatic disc to optimize esthetics and wear resistance. Bonding with a dual-cure MMA-based adhesive ensured a secure interface exceeding ISO retention standards. Polishing protocols achieved a surface roughness below the biofilm threshold of 0.2 μm, enhancing long-term hygiene maintenance and patient comfort.

At the six-month follow-up, the patient reported full satisfaction in terms of comfort, esthetics, and masticatory function, without any occlusal instability or mucosal irritation. These findings corroborate the clinical reliability of subtractively milled complete dentures and align with current clinical and systematic evidence supporting their precision, strength, and biocompatibility [4,5,10,17].

Despite these favorable results, limitations remain. Intraoral scanning of edentulous arches still requires operator skill and patient cooperation, particularly to capture vestibular anatomy without distortion. The initial cost of the CAD/CAM infrastructure and the learning curve associated with design software may also restrict accessibility in some clinical settings. Nonetheless, once implemented, digital workflows have the potential to augment reproducibility, optimize maintenance processes, and expedite the duplication of prostheses, especially for patients with specific medical requirements or limited time availability.

In summary, this case report highlights a reproducible, evidence-based digital workflow that integrates functional impression protocols and virtual design tools to deliver a high-quality maxillary complete denture. Its successful outcome supports the notion that digital prosthodontics can coexist with conventional techniques when selectively combined to enhance clinical precision and patient experience. As digital workflows become increasingly standardized, such approaches may serve as reliable models for clinicians seeking to implement digital denture fabrication in daily practice.

## 5. Conclusions

In this clinical case, the use of a fully digital workflow for fabricating a maxillary complete denture led to excellent clinical outcomes, including stable retention, accurate occlusion, and high-quality surface adaptation. These results can be attributed to the integration of border-molded functional impressions, virtual articulation, and subtractive CAD/CAM manufacturing using high-performance PMMA. The patient expressed complete satisfaction with the prosthesis, observing enhancements in functionality, aesthetics, and overall comfort, which positively influenced the quality of life.

Although the initial investment in digital infrastructure was substantial, the workflow proved efficient by reducing clinical chair time, minimizing the need for post-insertion adjustments, and improving communication between the clinical and laboratory teams. These advantages suggest that a structured digital protocol may be cost-effective and clinically advantageous in routine prosthodontic practice.

The successful outcome of this case reinforces current evidence supporting the use of CAD/CAM technology in complete denture fabrication. However, long-term clinical studies with larger patient samples remain necessary to validate the durability, biological behavior, and patient-reported outcomes of fully digital prostheses over time.

## Data Availability

The original contributions presented in this study are included in the article. Further inquiries can be directed to the corresponding author.
